# Accuracy of Point of Care Ultrasound in Assessment of Traumatic Eye Injuries: Quaternary Epitope Insights in Uricase Immunogenicity

**DOI:** 10.31661/gmj.v14i.3635

**Published:** 2025-03-16

**Authors:** Fatemeh Mohammadi, Hassan Amiri, Bahareh Seyedsalehi, Saeid Gholami Gharab, Mobin Naghshbandi, Manizhe Nasirizade, Samira Vaziri

**Affiliations:** ^1^ Department of Emergency Medicine, School of Medicine, Iran University of Medical Sciences, Tehran, Iran; ^2^ Emergency Medicine Management Research Center, Health Management Research Institute, School of Medicine, Iran University of Medical Sciences, Tehran, Iran; ^3^ Student Research Committee, School of Medicine, Iran University of Medical Sciences, Tehran, Iran; ^4^ Instructor of Nursing, Department of Medical Surgical Nursing, School of Nursing and Midwifery, Birjand University of Medical Sciences, Tehran, Iran

**Keywords:** Eye, Trauma, Point of Care Ultrasound (POCUS), Accuracy

## Abstract

**Background:**

A few studies have been conducted to assess the accuracy of point of care ultrasound in traumatic eye injuries. In the present study we aimed to examine the diagnostic value of Point of Care Ultrasound to assess eye injuries resulting from trauma.

**Materials and Methods:**

This observational study was performed on 221 consecutive patients with ocular trauma who admitted to emergency department of two teaching hospitals in 2016. On admission, all patients underwent ocular bedside ultrasonography to reveal ocular defects resulting from trauma. The diagnostic results of Point of Care Ultrasound were compared to the findings of clinical assessment of ophthalmologist as the gold standard.

**Results:**

Overalls, 13 lesions (5.9%) were revealed as ocular pathological lesions following trauma including retinal detachment in 6 cases, foreign body in 6 cases, and vitreous hemorrhage in one case. In this regard, Point of Care Ultrasound has a sensitivity of 86.7%, specificity of 94.7%, positive predictive value of 54.2%, negative predictive value of 98.9%, and an accuracy of 94.1%. The agreement coefficient between ultrasound and expert clinical assessment was 0.64 indicating an acceptable degree of agreement. (P0.001).

**Conclusion:**

Along with clinical assessment, Point of Care Ultrasonography of eye can accurately assess traumatic eye lesions.

## Introduction

In contemporary medical practice, imaging modalities such as Computed Tomography (CT) and Ultrasonography (US) have gained paramount importance, with an expanding role for Point-of-Care Ultrasound (POCUS) as an efficient diagnostic method, particularly in the management of multiple trauma patients, including those with eye trauma [1].

Despite the invaluable expertise of ophthalmologists, their clinical assessments are encumbered by several limitations, notably the inability to visualize lesions in the lens and cornea, as well as challenges posed by severe soft tissue injuries around the eye and decreased levels of consciousness [2][3]. Ocular trauma often leads to lodged foreign bodies, corneal edema, hyphoema, and vitreous hemorrhage [4]. POCUS has emerged as a rapid, safe, portable, and repeatable technique for detecting soft tissue lesions of the eye and orbit, presenting a promising diagnostic avenue in these scenarios [5][6]. The clinical significance of POCUS extends beyond just identification, as it also aids surgeons in planning appropriate surgical interventions for eye trauma patients [7][8]. Ocular ultrasonography serves a profound role in the assessment of both blunt and penetrating traumas affecting various ocular components. In essence, A-scan and B-scan ultrasonography enable the evaluation of what may not be observable or traceable through conventional clinical or ophthalmologic examinations [9][10].

While the diagnostic capabilities of ultrasonography in assessing eye traumas have been less extensively discussed, previous studies have begun to explore its sensitivity in this context. Hence, aligning with existing research, our present study aims to further examine the diagnostic value of ultrasonography in evaluating eye injuries resulting from traumas.

## Materials and Methods

### Study Design and Setting

This study is an observational cross-sectional study conducted at the Emergency Department of Haftom-Tir hospital in Tehran, Iran. This hospital serves as a tertiary trauma center with a high volume of over 70,000 admissions annually. The study was ethically approved by the Ethics Committee of Iran University of Medical Sciences, and written informed consent was obtained from the participating patients or their relatives.

### Participants

The study included patients who were referred to the Emergency Department due to blunt or penetrated ocular trauma. The inclusion criteria comprised ocular trauma patients over 18 years who consented to participate. Exclusion criteria involved patients with hemodynamic instability, those unable to cooperate for ultrasonography, or those who did not consent to participate in the study.

### Procedure

After initial surveys, ultrasound examinations were performed using a linear probe with minimal pressure on the eye within 30 minutes of the patients’ arrival. The types of ocular injuries were recorded, and the patients were then examined by an ophthalmologist. The ophthalmologist’s report served as the gold standard for assessing ocular injuries. Patients were followed up for one month, and the results of ultrasonography and clinical exams by the ophthalmologist were compared using tables and statistical methods.

### Ultrasound Examination

The ultrasound examinations were performed by an experienced emergency physician (ophthalmologist) with formal training in ocular ultrasound imaging. This physician had completed a certification course in emergency ultrasonography and had over three years of practical experience with trauma patients. Examinations were conducted using the Edge II Ultrasound system (Fujifilm Sonosite) with a 5 to 10 MHz linear transducer. The operator applied minimal pressure to the closed eyelid to avoid further ocular damage, following a protocol for standardized ocular ultrasound in trauma patients. The patient remained in the supine position to ensure immobilization, and all exams were completed within 30 minutes of the patient’s arrival.

### Data Analysis

Statistical analyses were carried out using SPSS software version 18. Quantitative variables were presented as mean ± standard deviation and summarized by frequency (percentage) for categorical variables. Statistical tests such as t-test, Mann-Whitney U test, and chi-square test were used for comparisons. Additionally, diagnostic values, including sensitivity and specificity of the tools, were calculated using specific formulas. To evaluate the concordance between ultrasonographic findings and clinical assessments, agreement percentage was calculated. This statistical measure was chosen to assess the level of agreement simply. Concordance was categorized based on the value as slight (0.01-0.20), fair (0.21-0.40), moderate (0.41-0.60), substantial (0.61-0.80), or almost perfect (0.81-1.00). Additionally, sensitivity, specificity, positive predictive value, and negative predictive value were determined to assess the diagnostic accuracy of the ultrasonography. Furthermore, receiver operating characteristic (ROC) curve was presented. The level of statistical significance was set at less than 5%.

## Results

In this study, a total of 221 cases of eye trauma were evaluated. The patient age range was 15 to 36 years, with 60.2% being male. Examining the underlying causes of trauma, 6.8% resulted from disputes, 69.2% from car accidents, 14.0% due to occupational hazards, and 1.4% from penetrating trauma (Table-1). Of these incidents, 13 cases (5.9%) were identified as having ocular pathological lesions post-trauma by ophthalmologist. These included retinal detachment in 6 cases, presence of a foreign body in 6 cases, and vitreous hemorrhage in one case (Table-2).

**Figure-1:**
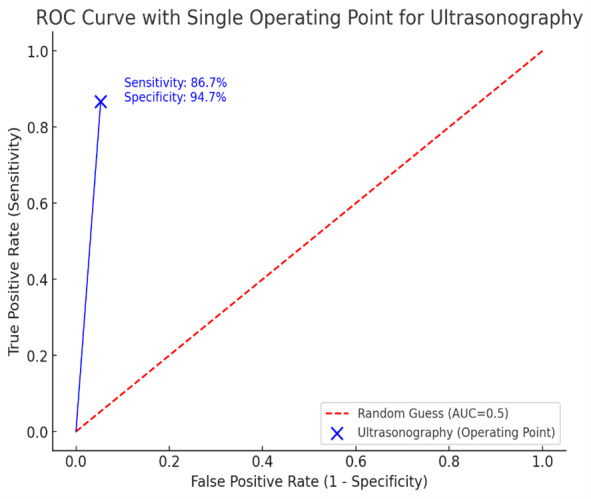
Figure 1. . the ROC curve with a single operating point for ultrasonography, based on the provided sensitivity (86.7%) and specificity (94.7%). The blue marker indicates the diagnostic performance of ultrasonography at this threshold, with the diagonal red line representing a random guess (AUC = 0.5). This point shows high sensitivity and specificity, suggesting good diagnostic accuracy at this specific threshold.

In terms of diagnostic efficacy, ultrasonography demonstrated a sensitivity of 86.7%, a specificity of 94.7%, a positive predictive value of 54.2%, a negative predictive value of 98.9%, and an overall accuracy of 94.1% (Figure-1). The concordance between ultrasonographic findings and clinical assessments was substantial, with an agreement value of 0.64, indicating a high level of agreement between these two diagnostic methods (P<0.001).

## Discussion

**Table:** Table1. Underlying causes of ocular trauma

Cause of Trauma	No of cases	Percentage
Dispute	15	6.8
Car Accidents	153	69.2
Occupational Hazards	31	14
Penetrating Trauma	3	1.4
Other	19	8.6

**Table:** Table2. Types of ocular trauma lesions diagnosed by ultrasonography

Type of Lesion	No of cases
Retinal Detachment	6
Foreign Body	6
Vitreous Hemorrhage	1
Total	13

Ultrasonography has become an indispensable tool in the realm of emergency medicine, particularly for evaluating patients with traumatic eye injuries. Its widespread use as a quick, portable, and non-invasive diagnostic tool is universally acknowledged. [11]. In emergency scenarios, where every second counts, ultrasonography’s role in facilitating rapid and definitive diagnoses is unparalleled, especially for patients suffering from traumatic ocular impairments. In many emergency departments, the initial assessment of eye injuries relies heavily on clinical examinations. While these are foundational in the diagnostic process, they depend greatly on the attending physician’s expertise and experience. Clinical assessments, although valuable, have limitations, especially when it comes to internal ocular structures or cases where symptoms are subtle or masked. Herein lies the advantage of ultrasonography: it transcends these limitations, providing a more comprehensive view of the eye’s internal condition, which is often critical in diagnosing complex traumatic injuries.

Compared to other imaging modalities like CT scans or MRI, ultrasonography stands out for its immediacy and accessibility. CT scans and MRI, though highly accurate, are often resource-intensive and not always immediately available, particularly in resource-limited settings. These methods can also be time-consuming, leading to potential delays in diagnosis and treatment. Ultrasonography, on the other hand, is relatively more accessible and can be conducted at the patient’s bedside, offering immediate insights into the patient’s condition [12]. This immediacy is crucial in trauma cases where rapid decision-making can significantly influence patient outcomes.

Our study’s findings indicate a higher incidence of ocular trauma in male patients and a notable prevalence in the middle-aged demographic. This trend suggests a need for targeted preventive measures and awareness programs, especially in high-risk occupations and activities prone to accidents. Additionally, the study’s results underscore the necessity of incorporating advanced diagnostic tools like ultrasonography in standard emergency care protocols.

The superiority of ultrasonography in diagnosing traumatic ocular lesions, as demonstrated in our study, is supported by a wealth of research. Haghighi et al.’s study [13] revealed ultrasonography’s high sensitivity and specificity in ocular trauma assessment (Sensitivity and specificity of ultrasonography were 84.6% (95% Cl: 53.7-97.3) and 98.3% (95% Cl: 93.3- 99.7), respectively.), with an excellent agreement with orbital CT scans (Cohen’s kappa coefficient of 0.83 (95% Cl: 0.66-1.0; P<0.0001)). Such findings are pivotal in validating ultrasonography as a reliable alternative to more invasive diagnostic methods. Similarly, the systematic review by Vrablik et al. [14] highlighted the efficacy of bedside ocular ultrasonography, especially in diagnosing retinal detachment, further supporting its utility in emergency ophthalmic care. Some studies highlights the high diagnostic accuracy of ocular ultrasonography for detecting retinal detachment in emergency settings. In Blaivas et al. (2002), emergency physicians achieved perfect sensitivity and specificity (100%) after brief training, with computed tomography or ophthalmologic evaluation as the reference [14]. Shinar et al. (2011) reported sensitivity of 97% and specificity of 92% among residents trained with a 30-minute lecture, using unblinded ophthalmologic exams as the standard. Yoonessi et al. (2010) found a sensitivity of 100% and specificity of 83% among acutely symptomatic patients [14]. Across studies, the area under the ROC curve ranged from 0.943 to 1.00, indicating excellent diagnostic performance. The combined area under the curve was 0.957, affirming the reliability of bedside ultrasonography for retinal detachment diagnosis in emergency departments [14].

The study by Zvornicanin et al. [15] not only emphasized ultrasonography’s role in confirming suspected diagnoses but also its potential in altering initial management plans based on new findings, showcasing its impact on patient treatment trajectories.

The rise of point-of-care ultrasound (POCUS) in the last two decades, encompassing ocular ultrasound, marks a significant advancement in emergency medicine [9]. POCUS has revolutionized patient assessment, particularly in scenarios where traditional examination methods are limited. For instance, in severe trauma cases with substantial eyelid swelling, clinical examination might be insufficient to assess the eye’s condition fully. Ultrasonography steps in as a critical tool, enabling assessment of internal ocular structures and facilitating diagnoses that would otherwise be challenging [10]. Its capabilities extend beyond routine assessments, aiding in the diagnosis of complex conditions like lens dislocations, hyphema, retinal detachment, and the presence of foreign bodies. Moreover, its utility in evaluating the zygomatico-orbital complex and diagnosing orbital fractures adds another dimension to its applicative value in trauma care [14].

## Conclusion

This study highlights the diagnostic value of bedside ultrasonography for ocular trauma, showing high sensitivity (86.7%), specificity (94.7%), and overall accuracy (94.1%). Ultrasonography indicated a strong ability to identify and exclude ocular lesions, supported by a high negative predictive value (98.9%). The substantial agreement (0.64) between ultrasound findings and ophthalmologic assessments underscores its reliability and potential as an effective diagnostic tool in emergency settings. These results suggest that ultrasonography can serve as a valuable complement to clinical evaluation, especially in trauma centers where timely diagnosis is essential.

## Conflict of Interest

The authors declare no conflict of interest concerning this study.
